# The Effect of Mouth Breathing on Facial Anthropometry

**DOI:** 10.1055/a-2625-9444

**Published:** 2025-07-08

**Authors:** Wijana Hasansulama, Shinta Fitri Boesoirie, Fitri Septiani

**Affiliations:** 1 Department of Otorhinolaryngology – Head and Neck Surgery, Faculty of Medicine, Universitas Padjadjaran and Dr. Hasan Sadikin General Hospital, Indonesia

**Keywords:** mouth breathing, cephalometry, mandibular angle, retrognathic

## Abstract

**Background:**

Breathing can occur either through the nose or mouth. Mouth breathing is the process of breathing through the mouth alone or mostly through the mouth for more than 6 months. Mouth breathing can affect facial development. This study aims to look at the effect of mouth breathing on facial anthropometry.

**Methods:**

This study used a case–control design conducted during March to September 2024 at Dr. Hasan Sadikin Hospital, Bandung, on subjects aged 7 to 23 years who were divided into two groups, namely mouth breathing and nasal breathing. Data were obtained from filling out the MBD-MBS (Mouth Breathing in Daytime and Mouth Breathing during Sleep) questionnaire, taking lateral cephalometric photographs, and cephalometric measurements using the WebCeph application. Statistical analysis was performed with SPSS software using the chi-square and Mann–Whitney tests.

**Results:**

There were significant differences in angular parameters between the two groups, namely the Sella-Nasion to Gonion-Gnathion (SN.GoGn) angle ( *p* = 0.029), the Frankfort mandibular angle (FMA; *p* = 0.023), and the mandibular plane to palatal plane (MP.PP) angle ( *p* = 0.012); the Articulare-Gonion-Menton (ArGoMe) angle was greater in the oral breathing group ( *p* = 0.003). The linear parameter values in both groups were not different ( *p* > 0.05).

**Conclusion:**

Mouth breathing affects facial anthropometry, resulting in an increase in retrognathic mandibular and maxillary angles.

## Introduction

Breathing occurs through both the nose and mouth, with the nose primarily responsible for warming, humidifying, and filtering inhaled air. Nasal breathing engages the diaphragm, facilitating diaphragmatic breathing, which enhances air delivery to the alveoli in the lungs. 1 2

Mouth breathing is defined as breathing predominantly or exclusively through the mouth for over 6 months. This definition has varied across the literature; some researchers define it based on duration as noted above, while others characterize it by the degree of oral versus nasal airflow or associated functional impairments. Ramirez-Yanez describes it as an oral habit that becomes established when nasal breathing is consistently replaced by an oral-predominant breathing pattern. 2 Lin et al. further elaborate that mouth breathing can be classified as obligatory (resulting from nasal obstruction) or habitual (persisting after nasal obstruction is resolved). The incidence rate of mouth breathing ranges from 17% to 50%, with a prevalence of 50% to 56%. 3 The likelihood of mouth breathing in children increases from 20% at age 6 to 40% by age 12. This condition is linked to higher rates of snoring and sleep apnea, as well as alterations in bone structure and facial appearance.

Craniofacial development begins in the fourth week of pregnancy and continues until puberty, involving neural tissue, muscle, cartilage, bone, and functional spaces. At birth, neural tissue reaches 60% to 70% of adult size, while muscle tissue reaches 50%. As growth progresses, facial features become more prominent, reflecting cranial growth. 3 4 5

From 2 months of embryonic age to 22 years, head size undergoes significant changes. During the first 5 years, the cranium and facial proportions are relatively large. Between ages 6 and 12, the face elongates, and features like the eyes and nose become more proportional. From ages 13 to 22, facial shape changes dramatically due to puberty, with clearer maxillary contours and more prominent mandibles. 6 7

The impact of mouth versus nasal breathing on craniofacial development is a subject of ongoing debate. Normal nasal breathing is crucial for balanced craniofacial growth, as it supports essential functions like mastication and swallowing. 8 9

Craniofacial development is shaped by various factors, including ethnic and racial differences, gender, genetics, and age. Research by Farkas et al. 10 highlighted anthropometric variations in craniofacial features across different races, while Reksodiputro 11 noted significant differences in facial anthropometry between Javanese and Caucasian women. Indonesian ethnic origins are traced back to two primary migrations from the Austromelanesoid and Mongoloid races, leading to the Proto Malay and later the Deutero-Malay populations, which include diverse ethnicities such as Javanese, Sundanese, and Balinese. These groups are classified as Oriental, with distinct brachyfacial characteristics as noted by Enlow. 8 9

Brachyfacial can also be the result of physiological adaptation to chronic mouth breathing from an early age, which affects craniofacial growth and development. Previous studies have primarily examined the effect of mouth breathing on facial morphology in White populations. However, significant differences in craniofacial anthropometry exist between White individuals and those of Indonesian ethnicity. 5

Based on previous findings in other populations, we hypothesize that mouth breathing significantly alters facial anthropometry in Deutero-Malay individuals, specifically resulting in increased mandibular plane angles (Sella-Nasion to Gonion-Gnathion angle [SN.GoGn], Frankfort mandibular angle [FMA]), greater maxillomandibular divergence (mandibular plane to palatal plane angle [MP.PP]), larger gonial angles (Articulare-Gonion-Menton [ArGoMe]), and retrognathic positioning of both mandible and maxilla. 8

We further hypothesize that while the general pattern of these alterations will be similar to those observed in previously studied populations, the magnitude of these changes may differ due to the inherent brachyfacial characteristics of the Deutero-Malay population. This study, therefore, aims to quantitatively assess these specific craniofacial parameters in Deutero-Malay individuals with mouth breathing compared to nasal breathing controls, to determine whether respiratory mode influences facial anthropometry in this ethnic group in ways consistent with or different from other populations.

## Materials and Methods

This cross-sectional case–control study with prospective data collection compared facial anthropometry between mouth breathers (case group) and nasal breathers (control group). The study was conducted from March 2024 to September 2024 at the Department of Otorhinolaryngology – Head and Neck Surgery, Dr. Hasan Sadikin General Hospital, Bandung, Indonesia.

Participants were prospectively recruited and evaluated during the study period. The case group consisted of patients with established mouth breathing (present for >6 months), while the control group included age- and gender-matched subjects without mouth breathing. Both groups underwent identical assessment protocols designed specifically for this research.

The study population consisted of patients of Deutero-Malay race aged 7 to 23 years. Inclusion criteria for the mouth breathing group (case group) included (1) history of breathing predominantly through the mouth for more than 6 months as reported by patients or caregivers; (2) positive diagnosis of mouth breathing as determined by the Mouth Breathing Diagnostic Questionnaire (MBDQ) with a score of ≥4; (3) clinical confirmation through physical examination showing at least two of the following characteristics: lip incompetence at rest, elongated face, narrow maxillary arch, or high palatal vault. Inclusion criteria for the nasal breathing group (control group) included (1) history of normal nasal breathing; (2) MBDQ score <4; (3) absence of clinical signs of mouth breathing during examination. Both groups required intact dentition appropriate for age, with normal growth and development patterns.

Exclusion criteria for both groups were history of facial reconstruction, orthodontic treatment, facial trauma, craniofacial congenital anomalies, and genetic disorders.

The minimum sample size was calculated using the formula for unpaired numerical analytic studies with continuous variables.



where,

- *n* = required sample size per group
= standard normal variate for a significance level of 5% (1.96)
- *
Z _β_* = standard normal variate for power of 80% (0.84)
- *σ* = pooled standard deviation from previous studies
- *δ* = expected difference between groups (effect size)

The effect size estimates were derived from three key previous studies. Zheng et al. (2020) reported significant differences in mandibular plane angles (SN.GoGn) between mouth and nasal breathers with a pooled standard deviation of 4.7 degrees and a mean difference of 2.5 degrees. 5 Chambi-Rocha et al. (2018) observed differences in the FMA with a standard deviation of 5.2 degrees and a mean difference of 2.8 degrees. 12 Additionally, Souki et al. (2012) reported differences in MP.PP angle with a standard deviation of 4.9 degrees and a mean difference of 2.7 degrees. 13

Using the most conservative estimates (largest standard deviation of 5.2 degrees from Chambi-Rocha et al. and smallest expected difference of 2.5 degrees from Zheng et al.), 5 12 the calculation was





Therefore, a minimum of 58 subjects per group (total *n* = 116) was calculated to be necessary to achieve 80% power (β = 0.20) at a 5% significance level (α = 0.05) for detecting a clinically meaningful difference of at least 2.5 degrees in facial angular measurements between mouth and nasal breathers. This calculation also accounted for potential variability in measurements across different cephalometric parameters. However, to further strengthen the study and account for possible dropouts or unusable data, we included 60 subjects in each group, resulting in a total sample size of 120 participants.

Patient recruitment was conducted using a selective sampling method. Potential participants of the Deutero-Malay race aged 7 to 23 years were identified from the outpatient clinic of the Department of Otorhinolaryngology – Head and Neck Surgery at Dr. Hasan Sadikin General Hospital between March 2024 and September 2024. For the mouth breathing group, patients were selected based on clinical presentation and physician referrals for suspected chronic mouth breathing. For the nasal breathing group, we selected control subjects matched by age and gender from patients visiting the clinic for unrelated issues who had no history or clinical signs of mouth breathing. The selection process continued until we reached the calculated sample size of 60 subjects in each group.

All eligible patients underwent a comprehensive assessment that included a medical history review and physical examination, completion of the MBDQ, and lateral cephalometric radiography. Lateral cephalograms were obtained using an Orthopantomograph OP 200 D machine (Instrumentarium Dental, PaloDEx Group Oy, Finland).

Participants were classified into the mouth-breathing group or nasal-breathing group based on a two-step protocol. First, all subjects completed the Mouth Breathing in Daytime and Mouth Breathing during Sleep (MBD-MBS) questionnaire, which evaluates the presence and frequency of mouth breathing symptoms both during the day and sleep. Subjects were classified as mouth breathers if they reported habitual or predominant mouth breathing for more than 6 months, as indicated by positive scores on the screening questionnaire. Key symptoms included open mouth posture at rest, frequent snoring, drooling during sleep, morning dry mouth, and observed mouth breathing by parents. Second, a confirmation assessment was conducted through clinical examination by an otorhinolaryngologist, including the mirror test and water-holding test. Subjects were finally assigned to the mouth breathing group if they showed positive findings both on the questionnaire and clinical examination. Participants who had no such symptoms and negative clinical findings were assigned to the nasal breathing group.

All radiographs were taken with patients in a natural head position, teeth in occlusion, and lips relaxed. Cephalometric landmarks and measurements were analyzed using WebCeph software (AssembleCircle Corp., Republic of Korea).

The cephalometric analysis included the evaluation of various angular and linear measurements. The angular measurements assessed were SNA (Sella-Nasion-A point angle, measuring maxillary position relative to cranial base), SNB (Sella-Nasion-B point angle, measuring mandibular position relative to cranial base), and ANB (A point-Nasion-B point angle, measuring maxillo–mandibular relationship), along with SN.GoGn (measuring mandibular plane inclination), FMA (measuring mandibular plane angle to Frankfort horizontal), and SN.MP (Sella-Nasion to Mandibular Plane angle, another measure of mandibular plane inclination). Additionally, NS.GoGn (Nasion-Sella to Gonion-Gnathion angle), SN.PP (Sella-Nasion to Palatal Plane angle), SN.OP (Sella-Nasion to Occlusal Plane angle), MP.PP angle, and ArGoMe angle (measuring gonial angle) were analyzed. For linear measurements, the parameters evaluated included N-Me (Nasion to Menton, measuring anterior facial height), ANS-Me (Anterior Nasal Spine to Menton, measuring lower anterior facial height), Ar-Go (Articulare to Gonion, measuring ramus height), and S-Go (Sella to Gonion, measuring posterior facial height). The Mann–Whitney U test was used to compare cephalometric measurements between groups.

The study protocol was approved by the Health Research Ethics Committee, Faculty of Medicine, Universitas Padjadjaran, Bandung, Indonesia (number: 300/UN6.KEP/EC/2024). Informed consent was obtained from all participants or their legal guardians. Patient confidentiality and data privacy were maintained throughout the study.

## Results

Table 1 shows that the composition of gender and age in both study groups did not differ ( *p* > 0.05).

**Table 1:** Subject characteristics

Characteristics	Mouth breathers ( *n* = 60)	Nose breathers ( *n* = 60)	*p* -Value a
Gender			**0.58**
Male	27 (45%)	24 (40%)	
Female	33 (55%)	36 (60%)	
Age (years)			**0.248**
Mean (SD)	17.3 (5.3)	17.0 (4.5)	
Median (range)	19.5 (6–23)	19 (6–23)	
Weight (kg)			
Mean (SD)	56.2 (12.3)	55.8 (11.7)	0.74
Height (cm)			
Mean (SD)	161.5 (9.6)	162.3 (9.4)	0.61
BMI (kg/m ^2^ )			
Mean (SD)	23.4 (3.2)	23.1 (3.4)	0.82

Abbreviations: BMI, body mass index; SD, standard deviation.

a Gender was analyzed using the chi-square test. Age, weight, height, and BMI were analyzed using the Mann–Whitney test.

Table 1 compares the demographic and anthropometric characteristics between mouth breathers ( *n* = 60) and nose breathers ( *n* = 60), demonstrating no statistically significant differences between the two groups. The gender distribution was similar (45% male in mouth breathers vs. 40% in nose breathers, *p* = 0.58). The mean ages were also comparable (17.3 vs. 17.0 years, *p* = 0.248), indicating that most participants had already passed their peak period of craniofacial growth. Other baseline characteristics, including weight (56.2 vs. 55.8 kg, *p* = 0.74), height (161.5 vs. 162.3 cm, *p* = 0.61), and body mass index (BMI; 21.4 vs. 21.1 kg/m ^2^ , *p* = 0.82), showed no significant differences. These non-significant *p* -values (all >0.05) confirm that both groups were well-matched, suggesting that any observed differences in study outcomes are unlikely to be attributable to baseline demographic or anthropometric factors.

Table 2 shows the facial angular parameters in both groups.

**Table 2:** Relationship between angular parameters and mouth breathing and nasal breathing

Variable	Group		*p* -Value a
	Mouth breathing ( *n* = 60)	Nasal breathing ( *n* = 60)	
Angle (degrees)			
SNA	85.18 (76.63–91.72)	83.90 (77.65–92.89)	0.754
SNB	79.76 (70.16–85.46)	79.59 (74.02–92.89)	0.985
ANB	5.89 (−1.56–8.41)	4.29 (−1.56–8.41)	0.201
SN.GoGn	33.76 (22.35–49.32)	28.88 (18.19–45.78)	0.029
FMA	25.73 (16.50–40.09)	25.08 (13.44–35.74)	0.023
SN.MP	34.78 (25.72–49.32)	33.76 (19.65–45.78)	0.081
NSGn	69.38 (60–77)	68.27 (55–77)	0.098
SN.PP	8.22 (3.78–12.67)	7.70 (3.78–12.67)	0.297
SN.OP	14.98 (6–27)	14.40 (1.64–24.27)	0.178
MP.PP	25.28 (18.56–39.17)	21.18 (12.44–35.54)	0.012
ArGoMe	120.79 (109–54)	118.20 (104–51)	0.003

Abbreviations: FMA, Frankfort mandibular angle; MP.PP, mandibular plane to palatal plane; SN.GoGn, Sella-Nasion to Gonion-Gnathion; SN.PP, Sella-Nasion to palatal plane angle.

a Mann–Whitney test.

Table 2 compares facial angular parameters between mouth breathing ( *n* = 60) and nasal breathing ( *n* = 60) groups, revealing several statistically significant differences. While many measurements showed similar values between groups, four parameters demonstrated significant differences ( *p* < 0.05): SN.GoGn (33.76 vs. 28.88 degrees, *p* = 0.029), FMA (25.73 vs. 25.08 degrees, *p* = 0.023), MP.PP (25.28 vs. 21.18 degrees, *p* = 0.012), and ArGoMe (120.79 vs. 118.20 degrees, *p* = 0.003). These findings suggest that individuals with mouth breathing tend to have higher values for these specific facial angles compared to nasal breathers, potentially indicating differences in craniofacial development patterns. Other measured parameters (SNA, SNB, ANB, SN.MP, NSGn, SN.PP, and SN.OP) showed no statistically significant differences between the two breathing pattern groups.

Table 3 shows the facial linear parameters in both groups.

**Table 3:** Relationship between linear parameters and mouth breathing and nasal breathing

Variable	Group		*p* -Value a
	Mouth breathing ( *n* = 60)	Nasal breathing ( *n* = 60)	
Linear			
N-Me	19.82 (15.96–22.19)	19.80 (15.96–22.19)	0.854
ANS-Me	11.36 (9.01–12.69)	11.28 (9.01–12.69)	0.217
Ar-Go	7.71 (6.21–10.48)	7.99 (6.39–10.48)	0.297
S-Go	13.21 (11.03–16.83)	13.41 (11.13–16.83)	0.226

a Using the Mann–Whitney test.

Table 3 compares facial linear parameters between mouth-breathing and nasal-breathing groups, each with 60 participants, showing four measurements: N-Me, ANS-Me, Ar-Go, and S-Go. The data reveal similar values across both breathing groups, with N-Me measurements of approximately 19.8, ANS-Me around 11.3, Ar-Go between 7.7 and 8.0, and S-Go about 13.2 to 13.4. All *p* -values (ranging from 0.217 to 0.854) calculated using the Mann–Whitney test exceed 0.05, indicating no statistically significant differences in facial linear parameters between mouth breathers and nasal breathers.

## Discussion

This study compares 60 subjects who are mouth breathers with 60 nasal breathers. While no significant difference in gender distribution was observed between groups ( *p* > 0.05), it is important to note that this study did not conduct formal interaction analyses to determine whether gender or age moderates the relationship between mouth breathing and facial morphology. Previous research by Acharya et al. 14 reported no significant differences in craniofacial development between males and females with different breathing patterns, but their methodology differs from the current study and may not be directly comparable. The biological mechanisms underlying craniofacial changes in mouth breathers might be explained through Moss's functional matrix theory, which posits that surrounding soft tissue function influences bone growth. These mechanisms could potentially operate similarly across gender and age groups through altered head and neck posture; however, this remains speculative without formal statistical testing of interaction effects. Future research should employ two-way analysis of variance (ANOVA) or regression models with interaction terms to systematically evaluate whether demographic factors moderate the impact of breathing patterns on facial anthropometry.

It is important to highlight that the average age of subjects in both groups was 17 years, with a median age of 19 years, indicating that most participants had already completed the majority of their craniofacial growth. Previous research demonstrates that significant facial development and susceptibility to environmental influences such as mouth breathing predominantly occur between the ages of 6 and 12 years, during active growth phases. In older adolescents and young adults, the bones and facial structures are more mature, likely reducing the potential impact of mouth breathing on craniofacial morphology. Therefore, the observed differences in facial angles between mouth and nasal breathing groups in this study may reflect changes established during earlier growth phases, rather than ongoing effects. This contrasts with most previous studies that included younger children, where the effect of mouth breathing on facial development is typically more pronounced. A detailed consideration of this age factor is important for the accurate interpretation and comparison of our findings.

Moreover, mouth breathing impacts pressure and airflow dynamics in the oral cavity and nasal passages similarly for both sexes. These changes disrupt the pressure balance in craniofacial structures, affecting their growth direction based on fundamental physiological principles rather than hormonal differences. The study's findings challenge Premkumar's 7 theory that gender influences craniofacial growth, which suggests that females typically experience earlier cessation of craniofacial growth due to hormonal factors during puberty.

While our initial analysis did not detect statistically significant age-related differences in craniofacial anthropometry among our subjects aged 7 to 23 years, this finding must be interpreted with substantial caution. Our study design lacked stratification by developmental growth phases (prepubertal, pubertal, and postpubertal), which is critical when examining craniofacial development across this wide age range. The relatively small sample size further limited our ability to detect age-dependent variations that have been well-documented in longitudinal growth studies. Established research in craniofacial development has consistently demonstrated significant changes in facial proportions and skeletal relationships throughout childhood and adolescence, particularly during puberty. Future studies should incorporate age stratification and larger sample sizes to properly assess how age-related craniofacial development interacts with breathing patterns.

The lack of gender and age as confounding variables prompts further investigation into the biological mechanisms linking mouth breathing to facial anthropometry. Mummolo et al. propose that the consistent influence of head and neck posture changes due to mouth breathing may explain the universal effects observed on facial anthropometry across different demographics. 15

The analysis reveals significant differences in facial anthropometry between mouth breathers and nasal breathers, particularly in variables such as SN.GoGn, FMA, MP.PP, and ArGoMe. These findings suggest that mouth breathing influences mandibular angles and palatal planes, corroborating previous studies that report larger mandibular angles and posterior–inferior rotation of the mandible in mouth breathers. Chronic mouth breathing alters head and neck posture to facilitate airflow, resulting in a posterior–inferior rotation of the mandible. This leads to an increased SN.GoGn angle and a larger FMA, indicating a lower ratio of posterior to anterior facial height. Consequently, individuals who habitually breathe through their mouths demonstrate clockwise rotation of the mandible, leading to relative vertical growth of the anterior face compared to the posterior. 5 10

**Fig. 1:**
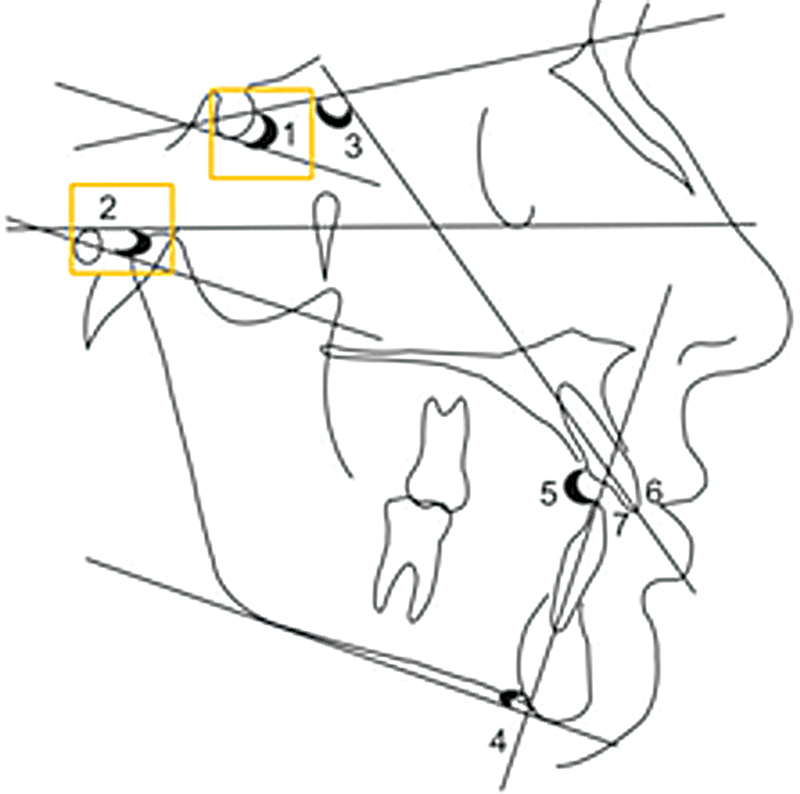
Measurement of angular cephalometric angles in lateral view. Angles : SN.GoGn (1), FMA (2), U1-SN (3), UMPA (4), U1-L1 (5), Overbite (6), and Overjet (7). FMA, Frankfort mandibular angle; SN.GoGn, Sella-Nasion to Gonion-Gnathion; U1-SN, Maxillary central incisor to Sella-Nasion; IMPA, Incisor Mandibular Plane Angle; U1-L1, Upper incisor to Lower Incisor.

Additionally, the study found that mouth breathers have narrower upper and lower dental arches. The larger FMAs observed align with findings from Masutomi et al., 16 which noted increased lower facial height and posterior mandible rotation in mouth breathers. This suggests that mouth breathing is associated with elongated facial growth patterns often linked to malocclusion ( Fig. 1 ).

Mouth breathing also lowers tongue position, reducing pressure on the palate and affecting maxillary development. This results in changes to MP.PP angles, consistent with Zheng et al.'s 5 findings that indicate larger MP.PP angles in mouth breathers are due to increased mandibular inclination. Furthermore, positional changes in the mandible due to airflow through the oral cavity lead to larger ArGoMe angles among mouth breathers. This reflects elongated faces where both maxillae and mandibles may adopt retrognathic positions, impacting aesthetic appearance and bite function, potentially leading to malocclusion.

**Fig. 2:**
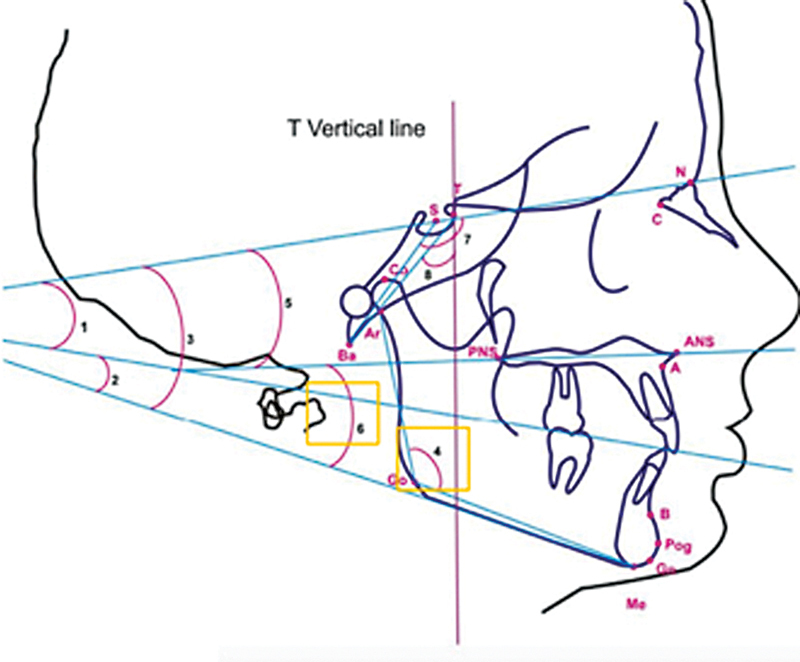
Measurement of angular cephalometric parameters in lateral cephalometry. Angles: SN.OP (1), OP-MP (2), SN-MP (3), ArGoMe (4), SN.PP (5), MP.PP (6), N-S-Ba (7), and Ba-T-Vert T (8). ArGoMe, Articulare-Gonion-Menton; MP.PP, mandibular plane to palatal plane; SN.OP, Sella-Nasion to occlusal plane; SN.PP, Sella-Nasion to palatal plane; OP-MP, Occlusal Plane to Mandibular Plane; SN-MP, Sella-Nasion to Mandibular Plane ; N-S-Ba, Nasion-Sella-Basion angle; Ba-T-Vert T, Basion to T point to vertical reference line through T angle.

Changes in posture and orofacial muscle function due to mouth breathing play a crucial role; perioral and buccal muscles undergo tonus alterations affecting mandibular and maxillary growth direction. This imbalance may reinforce tendencies for posterior–inferior rotation of the mandible, influencing nasomaxillary complex development. The combination of these factors results in measurable changes at craniofacial angles, particularly concerning mandibular position relative to palatal planes, reflecting long-term structural adaptations toward abnormal respiratory patterns, ultimately impacting overall facial anthropometry ( Fig. 2 ). 3 5 13

The SNA, SNB, and ANB angles are vital in cephalometric analysis for assessing maxillary–mandibular relationships relative to the skull base. In mouth breathers, SNA may show reduced values due to maxillary retrusion, although normal values can occur due to vertical face growth affecting maxillary positioning. Similarly, SNB angles tend to be smaller in mouth breathers, as the mandible often rests in a more retrusive position due to posterior rotation during mouth breathing, leading to a retrognathic appearance. 5 7

ANB, representing the difference between SNA and SNB, typically shows higher values (>4 degrees) in mouth breathers, indicating Class II malocclusion. This is attributed to either forward maxillary positioning or retruded mandibles resulting from posterior rotations. However, this study did not find significant differences in SNA, SNB, or ANB across groups, aligning with Masutomi et al.'s 16 findings, which also reported no statistically significant differences despite tendencies toward retrusive maxillae and mandibles among mouth breathers.

Previous studies have indicated a correlation between retrognathism and habitual mouth breathing; however, our findings do not fully support this, potentially due to methodological differences or variations in craniofacial development factors. 14

In terms of linear measurements like N-Me, ANS-Me, Ar-Go, and S-Go, no significant distinctions were observed between respiratory groups. This contrasts with other studies that reported increased anterior face heights among habitual mouth breathers. The observed discrepancies may suggest that the impact of oral respiration on vertical facial dimensions is inconsistent and influenced by individual growth patterns. 5

**Fig. 3:**
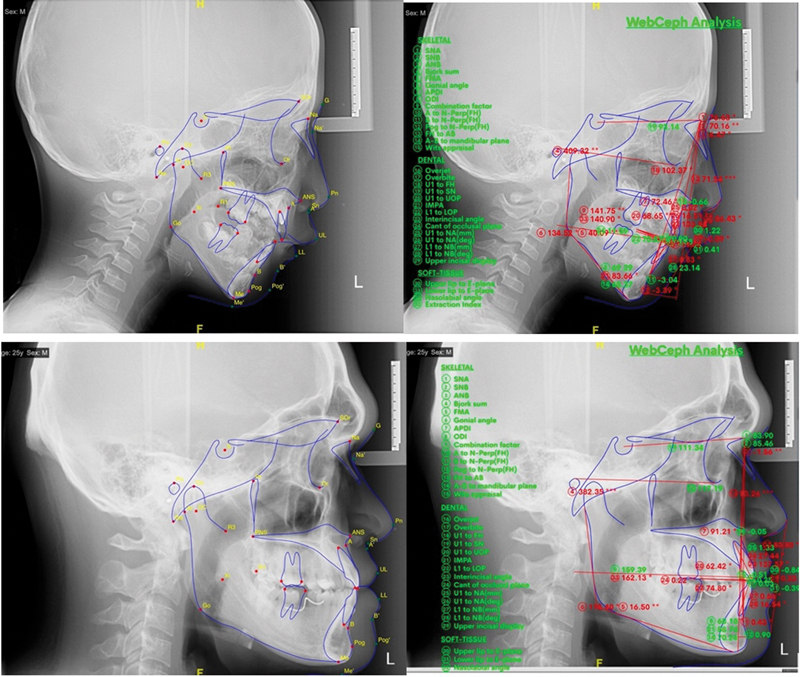
Lateral cephalometry in individuals with mouth breathing (top) and nose breathing (bottom).

Our analysis of linear variables focused on face heights revealed significant increases in total anterior face height and lower anterior heights among habitual mouth breathers, while noting declines in posterior heights. This suggests that skeletal maturity stages may influence height dynamics beyond respiratory modes ( Fig. 3 ). 5

Anthropometry changes associated with habitual oral respiration can lead to sleep-disordered breathing conditions such as sleep apnea, adversely affecting sleep quality and daytime concentration. Additionally, mouth breathing increases risks for pharyngitis and tonsillitis due to dry air exposure and reduced saliva function, which typically protects against infections and tooth decay. These complications can significantly impact quality of life if not addressed. 13 17

Ethnicity plays a crucial role in understanding human variations in craniofacial characteristics. While this study provides preliminary data on craniofacial morphology in Deutero-Malay individuals, further research with a larger, stratified sample is needed to establish comprehensive anthropometric profiles. Although brachyfacial forms are characteristic of this ethnicity, research by Aisy et al. 18 suggests that habitual mouth breathing may increase the risk of malocclusions due to suboptimal mandibular positioning. Our findings showed no significant differences in malocclusion outcomes across groups, consistent with Acharya et al.'s results.

### Conclusion

Mouth breathing significantly affects facial anthropometry. Individuals with mouth breathing exhibit a larger SN.GoGn angle, resulting in a more pronounced retrognathic mandible. The FMA is also greater, indicating a vertical growth pattern that can lead to malocclusion. Additionally, the MP.PP angle is larger, contributing to mandibular elongation and retrognathism of the maxilla, while the ArGoMe angle is increased, further promoting vertical facial growth and retrognathic features. However, linear measurements show no significant differences between mouth and nose breathing groups. It should be noted that, as most subjects were older adolescents or young adults, the impact of mouth breathing on craniofacial development observed in this study likely reflects long-term, cumulative effects rather than ongoing influences during active growth periods
